# Solubility and Dissolution Enhancement of Etoricoxib by Solid Dispersion Technique Using Sugar Carriers

**DOI:** 10.5402/2011/819765

**Published:** 2011-09-05

**Authors:** Abhisekh Das, Amit Kumar Nayak, Biswaranjan Mohanty, Satyabrata Panda

**Affiliations:** Department of Pharmaceutics, Seemanta Institute of Pharmaceutical Sciences, Orissa, Mayurbhanj 757086, India

## Abstract

The aim of the present study was to improve solubility and dissolution of the poorly aqueous soluble drug, etoricoxib by solvent evaporation technique using various sugar carriers, such as lactose, sucrose, and mannitol. Etoricoxib solid dispersions and their respective physical mixtures using lactose, sucrose, and mannitol were prepared in different ratios by solvent evaporation technique. The percent yield, drug content, saturation solubility, and in vitro dissolution of etoricoxib solid dispersions and physical mixtures were analyzed. Etoricoxib solid dispersions were characterized by FTIR spectroscopy, XRD, and DSC analysis. The FTIR spectroscopic analysis revealed the possibility of intermolecular hydrogen bonding in various solid dispersions. The XRD and DSC studies indicated the transformation of crystalline etoricoxib (in pure drug) to amorphous etoricoxib (in solid dispersions) by the solid dispersion technology. Both the aqueous solubility and dissolution of etoricoxib were observed in all etoricoxib solid dispersions as compared with pure etoricoxib and their physical mixtures. The in vitro dissolution studies exhibited improved dissolution in case of solid dispersion using lactose than the solid dispersions using both sucrose and mannitol. The in vitro dissolution of etoricoxib from these solid dispersions followed Hixson-Crowell model.

## 1. Introduction

Poorly aqueous soluble drugs are usually characterized by a low bioavailability due to less absorption, which is a major concern of pharmaceutical industries worldwide. Attempts to improve the solubility of these drug candidates have been performed by various approaches [1]. Among them, solid dispersion technique has attracted considerable interest as an efficient means of improving the dissolution rate, which increases the bioavailability of a range of poorly aqueous soluble drugs [2–4]. Fast and immediate drug dissolution from solid dispersions has been observed due to increased wettability, improved dispersibility of drug particles, and existence of the drug in amorphous form with improved solubility and absence of aggregation of drug particles using various hydrophilic carriers [1–4].

**Figure 1:**
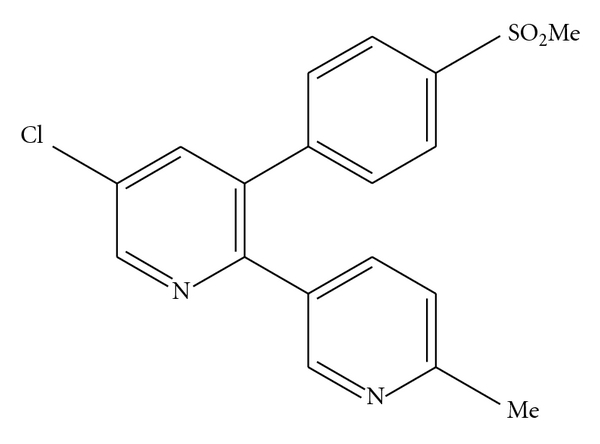
Chemical structure of etoricoxib.

Etoricoxib, 5-chloro-6′-methyl-3 [4-(methyl sulfonyl) phenyl]-2, 3′–bypyridine, is a highly selective second generation cyclooxygenage-2 (COX-2) inhibitor administered orally as an analgesic and nonsteroidal anti-inflammatory drug (NSAID) (Figure 1) [5]. Etoricoxib is an effective analgesic drug that has shown some improved efficacy versus traditional NSAIDs [6]. It is used in the treatment of rheumatoid arthritis, osteoarthritis, postoperative dental pain, chronic back pain, and acute gout [7, 8]. Moreover, recent studies evidenced its efficacy in patients with ankylosing spondylitis [6]. But it's very low aqueous solubility and poor dissolution can cause formulation problems and limit its therapeutic application by delaying the rate of absorption and the onset of action [7, 9]. Therefore, improvements in solubility and/or dissolution rate of etoricoxib may be achieved through the preparation of solid dispersions. In the literature, various solid dispersions of etoricoxib are reported for improving the dissolution of etoricoxib using various carriers like polyvinyl pyrrolidone K 30 (PVP K 30) [10], polyethylene glycol 4000 (PEG 4000) and polyvinyl pyrrolidone K 30 (PVP K 30) combination [11], Poloxamer 188 [12], and Gelucire 50/13, Compritol, and Sterotex K NF [13]. In the literature, various solid dispersions using sugars as carriers are reported [14–21]. In an investigation, Dha et al. have prepared etoricoxib solid dispersions using mannitol as carriers [22]. But, no attempt has been taken to prepare solid dispersion of etoricoxib using other common sugars like lactose and sucrose as carriers for solid dispersion of etoricoxib to improve the aqueous solubility and dissolution enhancement of etoricoxib till now. The enhancement of drug dissolution from sugar-based solid dispersions is mainly attributed to increase in surface area of drug exposed to large carrier molecules, increase wettability, and consequently solubility due to polar effect of sugars containing polar groups [1]. Again, sugars like lactose, sucrose, mannitol and so forth, are less expensive, easily available, and commonly used excipients in tablet and capsule formulations than other carriers used for the preparation of solid dispersion. So, the use of sugars as carriers for the preparation of solid dispersions of drug candidates may be able to reduce the production cost of the dosage forms, formulated using these low-cost sugar-based solid dispersions of drugs. Therefore, sugars as the suitable carriers for the preparation of solid dispersion were used in the present investigation. So, the aim of the present investigation is to prepare and characterize etoricoxib solid dispersion using various sugars like lactose, sucrose, and mannitol as carriers for improvements of solubility and/or dissolution of poor aqueous soluble drug, etoricoxib. Therefore, the objectives of this investigation are: (i) preparation of etoricoxib solid dispersions using lactose, sucrose and mannitol as carriers by solvent evaporation technique, (ii) characterization of prepared solid dispersions by Fourier transform infrared (FTIR) spectroscopy, X-ray diffraction (XRD), and differential scanning calorimetry (DSC) studies, and (iii) estimation of drug solubility and dissolution of prepared etoricoxib solid dispersions and comparing these data with that of pure drug and physical mixtures of drug carriers.

## 2. Materials and Methods

### 2.1. Materials

Etoricoxib was obtained as a gift sample from Cadila Healthcare Ltd., Moraiya, India. Lactose, sucrose, mannitol, potassium dihydrogen phosphate, and sodium hydroxide were purchased from S.D. Fine Chemicals Ltd., Mumbai, India. Ethanol (Bengal Chemicals and Pharmaceuticals Ltd., Kolkata, India) was used. All reagents were of A.R. graded. Double distilled water was used throughout the experiment.

### 2.2. Preparation of Etoricoxib Physical Mixtures Using Sugars

Physical mixtures of etoricoxib were prepared by mixing etoricoxib with lactose, sucrose, and mannitol in 1 : 1 and 1 : 5 ratios in a glass mortar by mixing for 10 minutes.

### 2.3. Preparation of Etoricoxib Solid Dispersions Using Sugars

Solid dispersions of etoricoxib were prepared by solvent evaporation technique using various sugars like lactose, sucrose, and mannitol as carriers in 1 : 1 and 1 : 5 ratios. Etoricoxib was dissolved in ethanol to get clear solution. Lactose, sucrose, and mannitol were dispersed as fine particles and the solvent was removed by evaporation on a water bath at 60°C. The dried mass was stored in desiccator until constant mass was obtained, crushed, and passed through sieve no. 22.

### 2.4. Determination of Percent Yield

The percent yield of etoricoxib solid dispersions was determined by using the following formula:

(1) $$\text{percent}\;\text{yield}=\frac{\text{Weight}\;\text{of}\;\text{prepared}\;\text{solid}\;\text{dispersion}}{\text{Weight}\;\text{of}\;\text{drug}+\text{carriers}}\times 100.$$

### 2.5. Determination of Percent Drug Content

Solid dispersions of etoricoxib (25 mg) were placed in 25 mL volumetric flask. Ethanol (10 mL) was added, mixed thoroughly using a rotating shaker for 1 hour. The volume was made up to the mark with ethanol. The solution was suitably diluted with ethanol and spectrophotometrically assayed for drug content at 233.5 nm using the following formula:

(2) $$\begin{array}{cc} & \text{percent}\;\text{drug}\;\text{content}\\ & =\frac{\text{Practical}\;\text{drug}\;\text{content}\;\text{in}\;\text{solid}\;\text{dispersions}}{\text{Theoretical}\;\text{drug}\;\text{content}\;\text{in}\;\text{solid}\;\text{dispersions}}\times 100. \end{array}$$

### 2.6. Determination of Saturation Solubility

To evaluate the increase in solubility of etoricoxib from solid dispersions, saturation solubility measurements were conducted and compared these data with that of pure etoricoxib and physical mixtures of respective ratios. The known excess samples (etoricoxib solid dispersions, physical mixtures, and pure etoricoxib) containing 10 mg equivalent weight of etoricoxib were added to 10 mL of phosphate buffer, pH = 7.4, and these samples were rotated at 20 rpm in a water bath (37 ± 0.5°C) for 48 hours. The samples were then filtered, suitably diluted, and analyzed by UV-VIS spectrophotometer (Shimadzu Corporation, Japan) at 284 nm wavelength.

### 2.7. Characterization

#### 2.7.1. Fourier Transform-Infrared (FTIR) Spectroscopy

Physicochemical characterization was performed using Fourier transform-infrared (FTIR) spectroscopy. For this purpose, samples were reduced to powder and analyzed as KBr pellets by using a FTIR spectrometer (Shimadzu Corporation, Japan).

#### 2.7.2. X-Ray Diffraction (XRD)

XRD patterns were recorded on an X diffractometer (Phillip PW 1130/00 diffractometer, The Netherlands), employing CuK*∞* radiation source operating at 30 mA and 40 kV. Samples were scanned from 6 to 40° 2*θ* at a scanning rate of 0.02° 2*θ* s^−1^.

#### 2.7.3. Differential Scanning Calorimetry (DSC)

The samples were analyzed by differential scanning calorimeter (Model DT-60, Shimadzu) at a constant scanning speed of 10°C min^−1^ from 0–300°C. The 5–7 mg samples were accurately weighed into solid aluminium pans without seals.

### 2.8. In Vitro Dissolution Studies

In vitro dissolution studies were performed in phosphate buffer (pH 7.4, 900 mL) at 37 ± 0.5°C, using USP XXIII apparatus (Electrolab, India) with a paddle rotating at 50 rpm. The samples (pure etoricoxib, etoricoxib physical mixtures, and etoricoxib solid dispersions) equivalent to 60 mg etoricoxib were subjected to dissolution. At fixed time intervals, samples (5 mL) were withdrawn and equal amount of fresh dissolution medium was added. Withdrawn samples were filtered (Whatman filter paper no. 41) and spectrophotometrically assayed for drug content at 284.0 nm wavelengths using a UV-VIS spectrophotometer (Shimadzu Corporation, Japan).

## 3. Results and Discussion

### 3.1. Percent Yield and Drug Content

Various etoricoxib solid dispersions using sugars like lactose, sucrose, and mannitol at different ratios (1 : 1 and 1 : 5) were prepared by solvent evaporation technique to increase the solubility and/or dissolution of poorly aqueous soluble drug, etoricoxib. The percent yield of various etoricoxib solid dispersions was within the range of 83.09 ± 2.13% to 91.18 ± 3.19% (Table 1). The percentage drug content in various newly prepared etoricoxib solid dispersions ranged from 95.75 ± 2.91% and 98.60 ± 2.13%, as reported in Table 1. This indicated that etoricoxib was uniformly distributed in all of these prepared solid dispersions. 

### 3.2. Saturation Solubility

The saturation solubility of pure etoricoxib, various newly prepared etoricoxib solid dispersions using sugars (lactose, sucrose, and mannitol), and their respective physical mixtures in phosphate buffer, pH 7.4 were measured. As etoricoxib has pH-dependent solubility [13], the change in pH may hamper the results during solubility measurement and in vitro drug release study. So, to maintain pH constant, phosphate buffer, pH 7.4 was used. Pure etoricoxib showed 78.48 ± 1.47 *μ*g/mL of saturation solubility. All of samples, both physical mixture and solid dispersions of etoricoxib using sugars, showed an increase in drug solubility (Table 2). All physical mixtures showed higher saturation solubility as compared with pure etoricoxib. Again, etoricoxib solid dispersions showed higher saturation solubility than their respective physical mixtures of drug and carrier. This might be attributable to an improvement of wetting of drug particles and localized solubilization by the water-soluble sugar carriers. In case of solid dispersions, the order of sugar carriers for increasing saturation solubility was lactose > mannitol > sucrose. 

### 3.3. Characterization

#### 3.3.1. FTIR Spectroscopy Analysis

**Figure 2:**
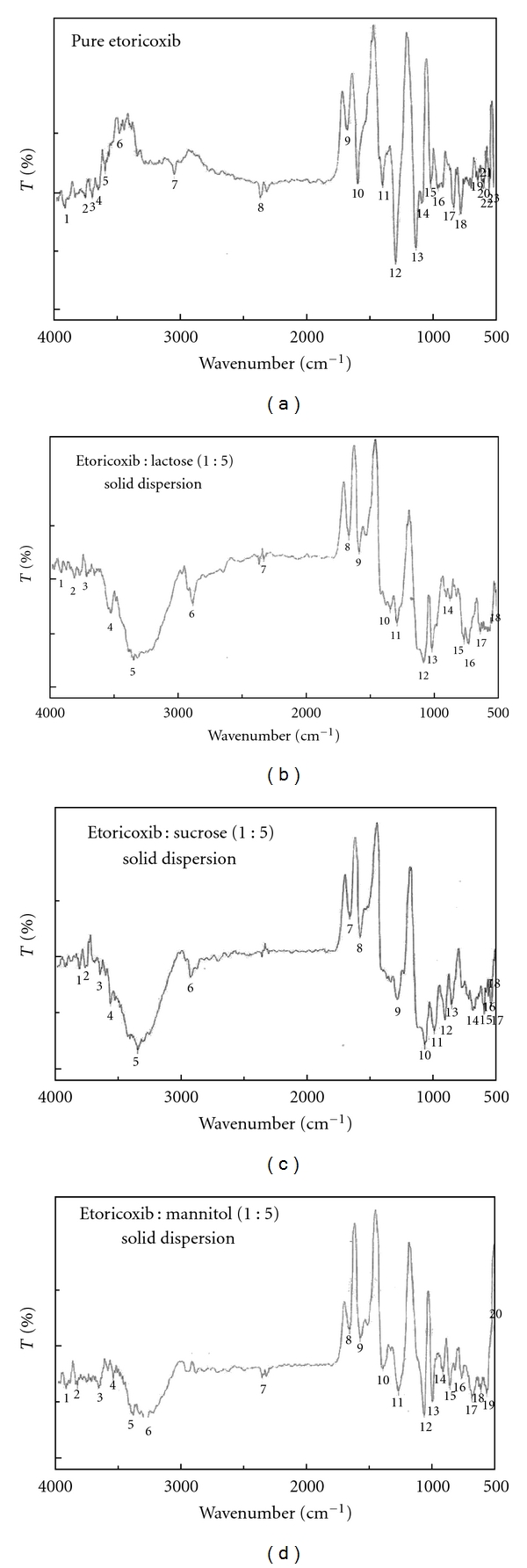
Fourier transform-infra red (FTIR) spectra of pure etoricoxib (a) and drug to carrier ratio, 1 : 5 etoricoxib solid dispersions using lactose (b), sucrose (c), and mannitol (d).

FTIR spectroscopy analysis was done to analyze physicochemical interactions between etoricoxib and sugar carriers in form of solid dispersions. FT-IR spectra of pure etoricoxib and various etoricoxib solid dispersions are shown in Figures 2(a), 2(b), 2(c), and 2(d). The FTIR spectra of pure etoricoxib showed characteristic peaks at 1592.9 cm^−1^ (C=N stretching vibration), 1430.0, 1299.4, 1136.8, and 1089.6 cm^−1^ (S=O stretching vibration) and 834.0, 777.1, and 639.22009 cm^−1^ (C–Cl stretching vibration). The FTIR spectrum of solid dispersions of etoricoxib using sugars like lactose, sucrose, and mannitol revealed a shift and slight broadening of 1291.3 cm^−1^ (in case of solid dispersions using lactose), 1290.6 cm^−1^ (in case of solid dispersions using sucrose), and etoricoxib and characteristic O–H stretching of vibration peaks, 3350.7 cm^−1^ (in case of solid dispersions using lactose), 3349.7 cm^−1^ (in case of solid dispersions using sucrose), and 3292.8 cm^−1^ (in case of solid dispersions using mannitol) of carriers. These observations might indicate the possibility of inter-molecular hydrogen bonding via the S=O group of etoricoxib and O–H group of sugar carriers.

#### 3.3.2. XRD Analysis

**Figure 3:**
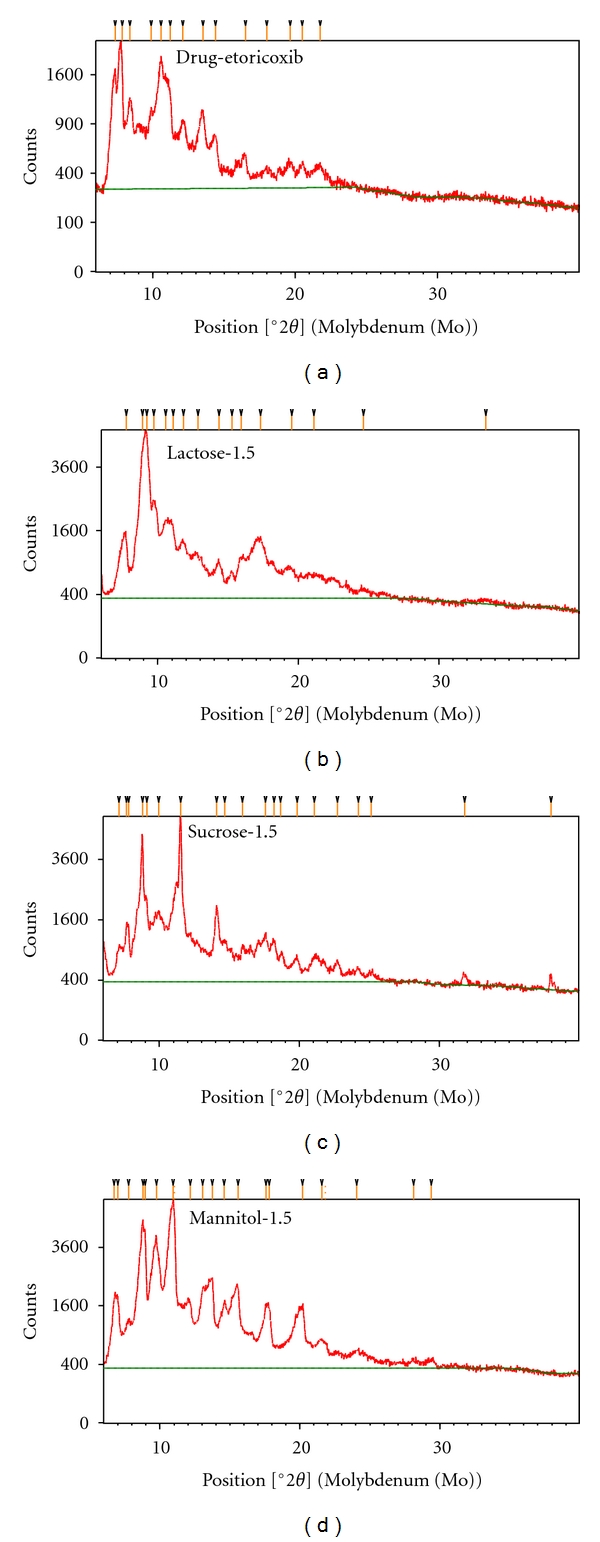
XRD pattern of pure etoricoxib (a) and drug to carrier ratio, 1 : 5 etoricoxib solid dispersions using lactose (b), sucrose (c), and mannitol (d).

The XRD patterns of pure etoricoxib and various newly prepared etoricoxib solid dispersions using sugars are presented in Figures 3(a), 3(b), 3(c), and 3(d). The characteristic peaks appeared in the XRD pattern of the pure etoricoxib at a diffraction angle of 9.65, 11.40, 14.41, 16.70, 19.62, 22.75, 24.10, 29.52, and 31.44° (2*θ*), suggesting that the drug is present as a crystalline state. The XRD pattern of etoricoxib solid dispersions using lactose, sucrose, and mannitol showed various characteristics peaks of pure etoricoxib with/without very small shifting. It was also observed that some peaks shown by pure etoricoxib were absent, and the intensity of peaks of these etoricoxib solid dispersions was found to be markedly reduced when compared to that of the pure etoricoxib. These observations indicate that the drug (etoricoxib) in solid dispersion was amorphous as compared to the pure drug.

#### 3.3.3. DSC Analysis

**Figure 4:**
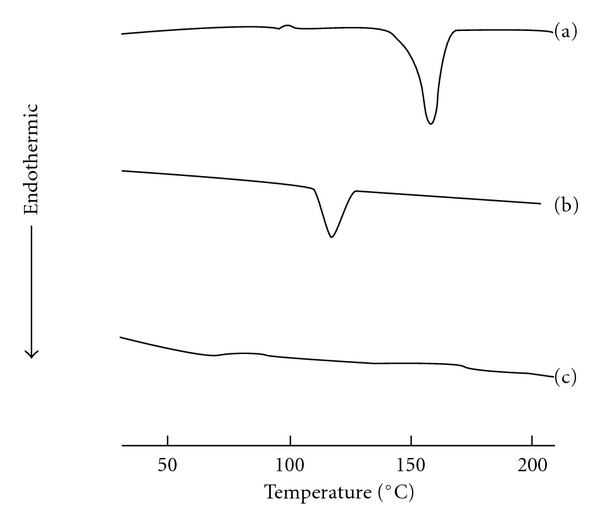
DSC thermogram of lactose (a), pure etoricoxib (b), and etoricoxib solid dispersions (c) using lactose (1 : 5).

DSC analysis was done for pure etoricoxib and solid dispersions of etoricoxib using lactose, the sugar carrier which showed higher saturation solubility than other sugars examined in this investigation. DSC pattern of etoricoxib solid dispersion using lactose, pure etoricoxib along with that of lactose are shown in Figure 4. The DSC thermogram of pure etoricoxib showed a sharp endothermic peak at 139.88°C, which was ascribed to drug melting. The DSC curve of lactose showed a sharp peak endothermic peak at 151.88°C, corresponding to the melting point of lactose. The peak temperature in solid dispersion was shifted slightly to lower temperature with respect to the drug, and there was a decrease in ΔH value of etoricoxib solid dispersion using lactose (−256.34 Jg^−1^) compared to the pure etoricoxib (−90.067 Jg^−1^). These phenomena could be attributed to the amorphous form of the etoricoxib in solid dispersion.

### 3.4. In Vitro Dissolution

**Figure 5:**
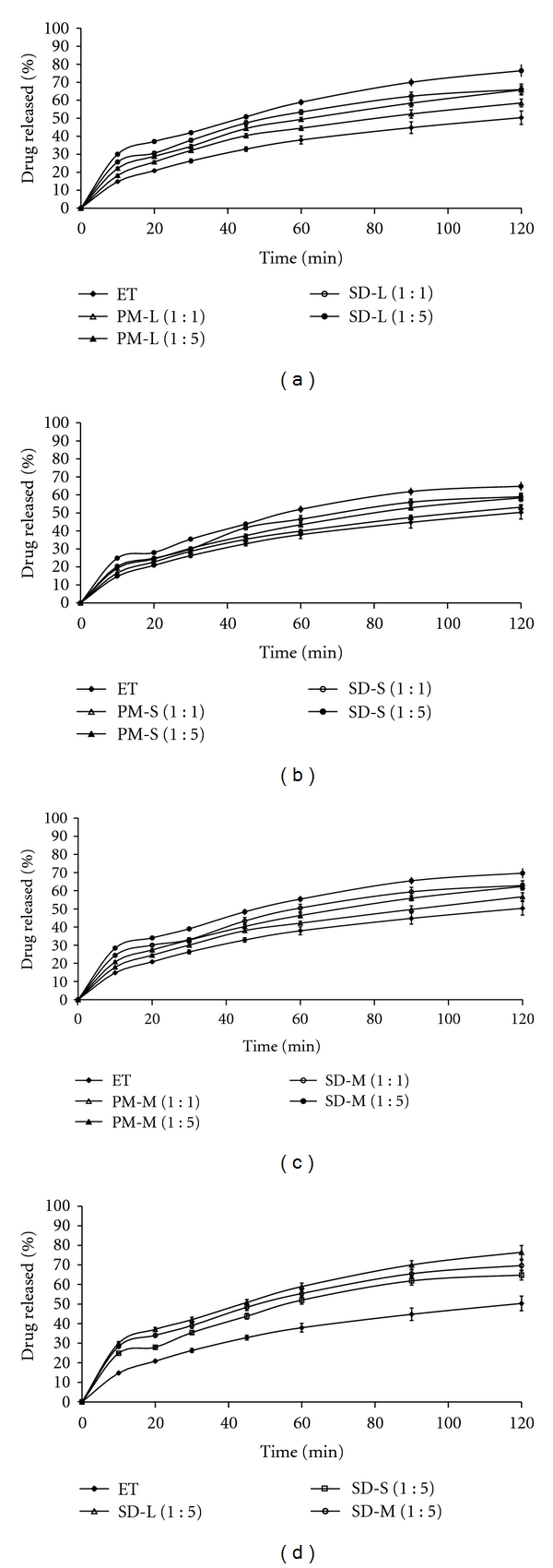
(a) Comparative in vitro dissolution profiles of etoricoxib solid dispersions using lactose [SD-L (1 : 1), SD-L (1 : 5)], etoricoxib-lactose physical mixtures [PM-L (1 : 1), PM-L (1 : 5)], and pure etoricoxib [ET] (Mean ± SD, *n* = 3). (b) Comparative in vitro dissolution profiles of etoricoxib solid dispersions using sucrose [SD-S (1 : 1), SD-S (1 : 5)], etoricoxib-sucrose physical mixtures [PM-S (1 : 1), PM-S (1 : 5)] and pure etoricoxib [ET] (Mean ± SD, *n* = 3). (c) Comparative in vitro dissolution profiles of etoricoxib solid dispersions using mannitol [SD-M (1 : 1), SD-M (1 : 5)], etoricoxib-mannitol physical mixtures [PM-M (1 : 1), PM-M (1 : 5)], and pure etoricoxib [ET] (Mean ± SD, *n* = 3). (d) Comparative in vitro dissolution profiles of etoricoxib solid dispersions using lactose [SD-L (1 : 5)], sucrose [SD-S (1 : 5), and mannitol [SD-M (1 : 5)], and pure etoricoxib [ET] (Mean ± SD, *n* = 3).

The in vitro dissolution profiles of the drug (etoricoxib), various solid dispersions using sugars, and their respective physical mixtures in phosphate buffer (pH = 7.4) for 120 minutes are shown in Figures 5(a) to 5(c). All of the physical mixture and solid dispersion samples showed improved dissolution of etoricoxib over pure etoricoxib. The enhancement of dissolution is mainly attributed to increased surface area of drug exposed to large carrier molecules, increased wettability, and accordingly solubility due to polar effect of sugars containing polar groups [1]. This also may be attributed to the higher hydrophilic sugar carriers, which can reduce the interfacial tension between the poorly aqueous soluble drug and the dissolution medium [23]. Again, all of the solid dispersion samples showed more improved etoricoxib dissolution than their respective physical mixture samples. This observation indicated that the increased dissolution of etoricoxib from solid dispersion due to presence of drug in amorphous state as compared to the physical mixtures and pure drug, where drug is present in crystalline state [24]. In case of etoricoxib solid dispersions, the order sugar carriers for increasing dissolution in phosphate buffer (pH = 7.4) was lactose > mannitol > sucrose (Figure 5(d)) and this observation is well correlated with the results of saturation solubility.

### 3.5. Drug Release Kinetics and Mechanism

In order to predict and correlate the mechanism and kinetics of etoricoxib release from etoricoxib solid dispersions using sugars, it is necessary to fit into a suitable mathematical model. The in vitro drug release data of these newly prepared solid dispersions were evaluated kinetically using various mathematical models like zero order, first order, Higuchi, Hixson-Crowell, and Korsmeyer-Peppas model [18, 25].

Zero-order model:

(3) $$F=K_{0}t,$$

where *F* represents the fraction of drug released in time *t*, and *K*_0_ is the apparent release rate constant or zero-order release constant. 

First-order model:

(4) $$\ln\left(1-F\right)=-K_{1\text{st}}t,$$

where *F* represents the fraction of drug released in time *t*, and *K*_1_ is the first-order release constant. 

Higuchi model:

(5) $$F=K_{\text{H}}t^{1/2},$$

where *F* represents the fraction of drug released in time *t*, and *K*_H_ is the Higuchi dissolution constant. 

Hixson-Crowell model:

(6) $$W_{0}^{1/3}-W_{t}^{1/3}=Kt,$$

where *W*_0_ and *W*_*t*_ represent initial mass, and mass remained at time *t*, respectively; *K*_HC_ is the rate constant.

Korsmeyer-Peppas model:

(7) $$F=K_{\text{P}}t^{n},$$

where *F* represents the fraction of drug released in time *t*, *K*_P_ is the rate constant, and *n* is the diffusional exponent; this indicates the drug release mechanism. 

**Figure 6:**
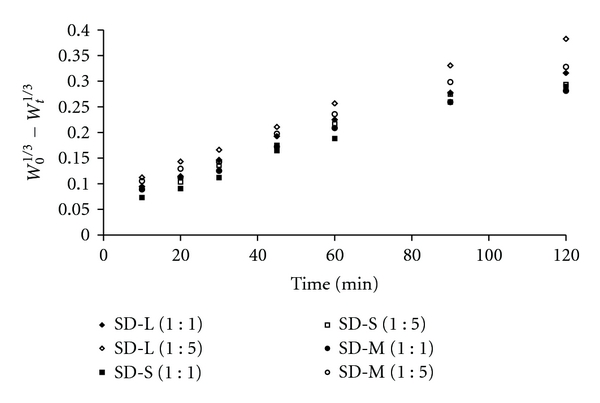
Hixson-Crowell's dissolution plots of etoricoxib solid dispersions using sugars SD-L (1 : 1), and SD-L (1 : 5): etoricoxib solid dispersions using lactose; SD-S (1 : 1), and SD-S (1 : 5): etoricoxib solid dispersions using sucrose; SD-M (1 : 1), and SD-M (1 : 5): etoricoxib solid dispersions using mannitol.

The results of the curve fitting into these above-mentioned mathematical models indicate the release behaviour of etoricoxib from these newly prepared solid dispersions (Table 3). When the correlation coefficients of these mathematical models for etoricoxib release were compared, it was found to follow Hixson-Crowell model with the best-fit correlation coefficient value (*R^2^* = 0.9839 to 0.9958). Again, a plot of *W*_0_^1/3^ − *W*_*t*_^1/3^ versus time using dissolution data was drawn and it was found linear (Figure 6) with all etoricoxib solid dispersions using various sugars like lactose, sucrose, and mannitol. This observation indicates that the dissolution of etoricoxib from these newly prepared solid dispersions occurred from discretely suspended or deposited (monodispersed) particles [26]. This might have also contributed to the enhanced dissolution rate of etoricoxib from solid dispersions using sugars as hydrophilic carriers.

## 4. Conclusion

Etoricoxib solid dispersions using various sugars like lactose, sucrose, and mannitol were successfully prepared by solvent evaporation technique. FTIR spectroscopy revealed the possibility of intermolecular hydrogen bonding in various solid dispersions. XRD and DSC observations indicated that the transformation of crystalline etoricoxib (in pure drug) to amorphous etoricoxib (in solid dispersions) by solid dispersion technology. The saturation solubility and in vitro dissolution studies showed a remarkable increase in both the solubility and dissolution of etoricoxib solid dispersions using sugars as compared with pure etoricoxib and their physical mixtures. The in vitro dissolution studies of all these newly prepared solid dispersions showed that the improved solubility and dissolution in case of solid dispersion using lactose than the solid dispersions using both sucrose and mannitol. The in vitro dissolution of etoricoxib from these solid dispersions was found to follow Hixson-Crowell model. Therefore, the solubility and dissolution of poorly aqueous soluble etoricoxib can be enhanced by the preparation of solid dispersions using sugars as hydrophilic carriers.

## Figures and Tables

**Table 1:** Percent yield and percent drug content of etoricoxib solid dispersions.

Solid dispersion type	Ratio	Percent yield (%)^$^	Percent drug content (%)^$^
Etoricoxib : lactose	1 : 1	88.13 ± 7.53	96.68 ± 1.99
	1 : 5	91.18 ± 3.19	98.39 ± 2.42
Etoricoxib : sucrose	1 : 1	85.43 ± 2.43	97.94 ± 2.50
	1 : 5	89.63 ± 3.62	98.60 ± 2.13
Etoricoxib : mannitol	1 : 1	83.09 ± 2.13	95.75 ± 2.91
	1 : 5	86.51 ± 2.15	97.32 ± 0.85

^$^Mean ± SD, *n* = 3.

**Table 2:** Saturation solubility of different etoricoxib solid dispersions along with pure etoricoxib and physical mixtures using same carriers (Mean ± SD, *n* = 3).

Type	Ratio	Saturation solubility (*μ*g/mL)^$^	
		Physical mixtures	Solid dispersions
	1 : 1	91.65 ± 4.95	124.28 ± 1.93
Etoricoxib : lactose	1 : 5	98.27 ± 5.04	142.26 ± 2.01
	1 : 1	84.91 ± 1.44	107.88 ± 2.87
Etoricoxib : sucrose	1 : 5	89.80 ± 4.12	120.42 ± 2.55
	1 : 1	90.50 ± 5.20	118.82 ± 2.01
Etoricoxib : mannitol	1 : 5	95.76 ± 6.67	131.98 ± 3.48

Pure etoricoxib	—	Saturation solubility (*μ*g/mL)^$^	
		78.48 ± 1.47	

^$^Mean ± Standard deviation, *n* = 3.

**Table 3:** Correlation coefficient (*R^2^*) values in the analysis of dissolution data of etoricoxib solid dispersions using sugars.

Mathematical models	Formulation codes*					
	SD-L (1 : 1)	SD-L (1 : 5)	SD-S (1 : 1)	SD-S (1 : 5)	SD-M (1 : 1)	SD-M (1 : 5)
Zero-order model	0.9462	0.9681	0.9276	0.9389	0.9276	0.9502
First-order model	0.9846	0.9871	0.9599	0.9689	0.9718	0.9807
Higuchi model	0.9892	0.9852	0.9756	0.9782	0.9714	0.9824
Hixson-Crowell model	0.9943	0.9958	0.9886	0.9882	0.9837	0.9897
Korsmeyer-Peppas model	0.9870	0.9807	0.9754	0.9718	0.9728	0.9739

*Formulation codes:

SD-L (1 : 1), and SD-L (1 : 5): etoricoxib solid dispersions using lactose

SD-S (1 : 1), and SD-S (1 : 5): etoricoxib solid dispersions using sucrose

SD-M (1 : 1), and SD-M (1 : 5): etoricoxib solid dispersions using mannitol.
